# Improving Prediction Accuracy for WSN Data Reduction by Applying Multivariate Spatio-Temporal Correlation

**DOI:** 10.3390/s111110010

**Published:** 2011-10-25

**Authors:** Carlos Carvalho, Danielo G. Gomes, Nazim Agoulmine, José Neuman de Souza

**Affiliations:** 1 Group of Computer Networks, Software Engineering and Systems (GREat), Federal University of Ceará, CEP 60455-760, Fortaleza, Brazil; E-Mails: danielo@ufc.br (D.G.); neuman@ufc.br (J.S.); 2 LRSM/IBISC Laboratory, University of Evry Val d’Essonne, 91020 Evry Courcouronnes CE 1433, France; E-Mail: nazim.agoulmine@iup.univ-evry.fr

**Keywords:** wireless sensor networks, multivariate correlation, data reduction

## Abstract

This paper proposes a method based on multivariate spatial and temporal correlation to improve prediction accuracy in data reduction for Wireless Sensor Networks (WSN). Prediction of data not sent to the sink node is a technique used to save energy in WSNs by reducing the amount of data traffic. However, it may not be very accurate. Simulations were made involving simple linear regression and multiple linear regression functions to assess the performance of the proposed method. The results show a higher correlation between gathered inputs when compared to time, which is an independent variable widely used for prediction and forecasting. Prediction accuracy is lower when simple linear regression is used, whereas multiple linear regression is the most accurate one. In addition to that, our proposal outperforms some current solutions by about 50% in humidity prediction and 21% in light prediction. To the best of our knowledge, we believe that we are probably the first to address prediction based on multivariate correlation for WSN data reduction.

## 1. Introduction

Wireless Sensor Networks (WSNs) consist of few or several sensor nodes which are resource constrained. Some sensor nodes gather data from external environments and send information such as temperature, humidity and light to the sink. The information is sent hop by hop (intermediate nodes) until the sink is reached. However, data traffic is a problem in WSN due to high energy consumption [1–3].

These sensors can be used in many applications such as event detection, location, monitoring and control [4]. Among these applications, environment monitoring is a very common scenario. Therefore, data gathering is periodical, generating a large amount of data traffic in the network.

In this scenario, the sensor nodes frequently send the same data gathered from a specific area. The overlapping of information sent to the sink causes waste of energy, which decreases the network lifetime. The problem is even worse when the number of deployed nodes increases (scalability), because data communication is responsible for most of the energy consumption in WSN [4–6].

**Figure 1.**
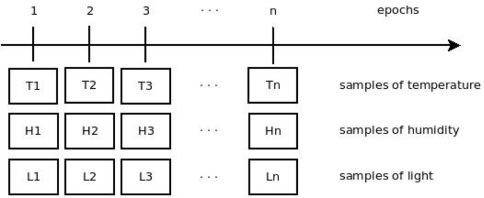
Operation of the monitoring system.

Figure 1 describes how the monitoring system works. Note that each sensor node gathers samples of a particular variable (such as temperature) and sends it to the sink at each cycle (epoch).

An energy efficient communication protocol helps improve the deployment of this type of network in environments such as vegetation and weather monitoring. The correlation between the data gathered by a sensor node and its neighbors, as well as the correlation between the data gathered by the sensor node itself over a given time [2] must be explored by efficient protocols to improve energy consumption. They are known as spatial and temporal correlation. When more than one variable in the correlation is taken into account, the approach is named multivariate correlation.

**Figure 2.**
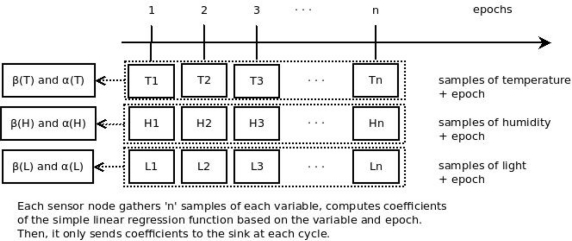
Operation of the monitoring system based on prediction proposed by current authors (simple linear regression).

The purpose of data prediction is to reduce data traffic to the sink. It has been adopted in several papers in the literature [7]. It helps to reduce the overall energy consumption of the network. An algorithm is embedded within the sensor node to calculate the coefficients of a linear regression function. These coefficients are named β and α, and represent a sequence of variable samples gathered by the sensor, such as temperature. Thus, the sensor node sends the coefficients to the sink, instead of sending the sequence of variables samples. When β and α arrive at the sink, they are used by the linear regression function embedded within the sink. Then the readings sequence is predicted by the monitoring system (Figure 2).

That approach usually takes into account the correlation of only one variable to be predicted (named dependent or response variable, e.g., temperature) and only one variable to predict the dependent variable (named independent or explanatory variable, e.g., time/epoch). However, the time variable is not the most correlated variable with others variables such as temperature, humidity and light.

Thus, the prediction adopted by current solutions, is sometimes not accurate. Consequently, the questions we address here are: “can we use the correlation between the variables gathered by the same sensor node to improve prediction accuracy?” and “is the multivariate prediction more accurate than published methods?”

**Figure 3.**
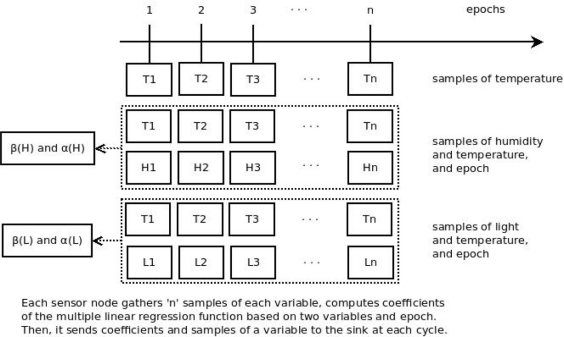
Operation of the monitoring system based on prediction proposed on this paper (multiple linear regression).

We propose a method that performs prediction of data based on multivariate correlation. In our method, we take into account the correlation between two readings of data gathered by the sensor node and also the time/epoch variable (Figure 3). Our method is different from current works which use the correlation between one variable gathered and the time variable.

## 2. Principles

In our approach we use a tree-based routing protocol to forward the data traffic from sensor nodes to the sink node, an approach similar to the one adopted by Li *et al.* [8]. To avoid spatial overlapping, each sensor node checks whether there is a degree of multivariate correlation between the packets previously sent by its neighbors. This is done before each sensor node sends the linear regression coefficients. Moreover, we also use the multivariate correlation method to avoid temporal overlapping in the same sensor node.

In this paper, simulations with simple and multiple linear regression functions are carried out to evaluate the prediction solution. For our solution, initially the correlation degree of the variables gathered by the sensor node is measured to decide which variable will be the independent one. Here in this paper, the Pearson’s coefficient (r) [9] in a real data trace indicates the strength of a linear relationship between two variables, e.g., if the variables are independent, Pearson’s coefficient is zero. We evaluate the energy consumption and prediction accuracy in every solution, in which the sensor nodes run simple linear regression (current solution) or multiple linear regression (our solution) function.

An original application to data collection without any prediction mechanism was developed. This application emulates a real gathering of temperature, humidity and light data. Then, the original version of this application is compared to three enhanced versions, where two use simple linear regression and one uses multiple linear regression. The prediction accuracy performance is evaluated by means of Residual Sum of Squares (SSerr) and coefficient of determination (R^2^).

## 3. Related Work

Goel and Imielinski [10] applied the concepts of MPEG compression to reduce energy consumption. They proposed a prediction based on a monitoring mechanism, called PREMON, which abstracts the data stream sent by sensor nodes to the sink as a video stream encoded by MPEG standard.

After PREMON, some works [8,11–14] have shown the feasibility of the use of spatial and temporal correlation to optimize the communication protocols in WSN. They use algorithms embedded within motes, in a distributed way, to reduce data transmission to the sink. These techniques reduce energy consumption and consequently increase the network lifetime.

Xu and Lee [15] proposed a localized prediction mechanism based on object tracking that reduces energy consumption due to hierarchy topology. According to Santini and Romer [13], sensor nodes in a distributed way are not able to operate, by itself, a data reduction system that can be as accurate as a centralized system. It uses statistics of the data history gathered by sensor nodes.

Matos *et al.* [7] proposed a simple linear regression to reduce data generated by sensor nodes which gather temperature from the external environment. They compared the prediction accuracy performance of the simple linear regression with prediction based on the average. The difficulty lies in the fact that prediction accuracy based on simple linear regression depends on only one variable, which in many situations, is not correlated with any other. The time variable is usually less correlated than other variables gathered in the field, such as temperature, humidity or light. Therefore, prediction errors tend to be higher, *i.e.*, less accurate. That paper is the closest to our proposed solution, but it performs prediction of user’s queries, instead of constantly performing stream predictions.

Seo *et al.* [16] carried out evaluations of some techniques for reducing the multivariate data traffic. These techniques are based on wavelet, sampling, hierarchical clustering and Singular Value Decomposition—SVD.

Silva *et al.* [17] reduced the multivariate dimensionality of data gathered by sensor nodes. The authors used Principal Component Analysis—PCA as a reduction technique in an air quality monitoring application. The algorithm identifies the more significant samples and then sends them to the sink. The highlight of that work is that the parameters’ performance, such as reduced data quality, energy consumption and delay, are taken into account in the experiments. Therefore, it is possible to observe the effects of applying the technique in multivariate data reduction. However, multivariate spatial correlation is not addressed. Also, there are few details about the solution operation, mainly about the error resulting from the dimensionality reduction procedure.

Multivariate spatial and temporal correlation is the key to solve problems of prediction accuracy and improve energy savings through data reduction techniques. The papers found in the literature have superficially addressed prediction accuracy, but it is an essential issue in WSNs.

This paper has the advantage (Table 1) of performing correlation analysis of variables gathered by sensor nodes before prediction is implemented. Also, the effects of using prediction based on multivariate spatial and temporal correlation in WSN were checked. Implementation details of our solution are highlighted, revealing the challenges of embed simple and multiple linear regression in WSN. In addition, we show when the use of prediction based on the multivariate correlation method is more appropriate, according to results.

Our work is inspired on these techniques and concepts (spatial and temporal correlation, data reduction and prediction), already known in the literature to address energy saving issues in WSN. However, we focus on the challenge of improving prediction accuracy of WSN data based on a multivariate correlation method.

## 4. Background

Several techniques have been defined to optimize energy consumption in applications for reducing data sent to the sink. The most common are compression, aggregation and fusion [1,4]. Such techniques are usually used without taking into account the multivariate spatial and temporal correlation of readings gathered by sensors nodes on field. However, many sensor nodes deployed on field are usually able to monitor more than one variable, and are thus called multisensors.

This section describes two concepts used by current works found in the literature, which we used in the conception of our solution. To the best of our knowledge, there is no other paper that uses multiple linear regressions to perform prediction and Euclidian distance to check correlation between neighbor sensor nodes readings, but we found papers such as that of Skordylis *et al.* [14] which use a technique adopted for spatial correlated data reduction by Pearson’s coefficient (r). Also, we found papers such as the one by Matos *et al.* [7] which uses a technique adopted for temporal correlated data reduction by simple linear regression. Next, we present these two concepts and the corresponding equations.

### 4.1. Pearson’s Coefficient

Pearson’s coefficient [Equation (1)] is used to identify the spatial correlation of the same variable between two sensor nodes [14]. But, it can also be used to identify the correlation between two variables of the same sensor node:

(1) $$r_{X_{1},X_{2}}=\frac{\sum \left(x_{1_{i}}-{\bar{X}}_{1}\right)*\left(x_{2_{i}}-{\bar{X}}_{2}\right)}{\sqrt{\sum {\left(x_{1_{i}}-{\bar{X}}_{1}\right)}^{2}*{\left(x_{2_{i}}-{\bar{X}}_{2}\right)}^{2}}}$$

where *r*_*X*_1_, *X*_2__ represents the relationship between two one-dimensional vectors *X*_1_ and *X*_2_, to be compared in terms of their correlation. They contain samples window of two variables, *X*_1_ = *x*_1_1__, … ,*x*_1_*i*__ and *X*_2_ = *x*_2_1__, … ,*x*_2_*i*__, where *i* = 1, … ,*n* and *n* is the number of samples. $\bar{X_{1}}$ and $\bar{X_{2}}$ represent the average of samples of each variable vector.

The coefficient *r* measures the degree of linear relationship between two one-dimensional vectors and its results can range from −1 to 1 (real numbers, e.g., 0.9 is highly correlated and −0.9 is also highly correlated and 0 is little correlated). There is a perfect linear relationship (two vectors are increasing or decreasing their values) when the correlation value is 1. On the other hand, there is a perfect inverse linear relationship (one vector increases its values while the other decreases its values) when correlation value is −1. There is no linear relationship between two vectors if the correlation value is 0 (zero).

Therefore, when coefficient (r) is close to the highest or lowest value (1 or −1), then the correlation between two vectors is high. Thus, we can calculate the spatial and temporal correlation of the readings of just one variable between two neighbor sensor nodes [14]. The problem is that we cannot calculate the multivariate spatial correlation by using this method, which is necessary for our solution. However, the next section shows how Euclidian distance is used to identify the multivariate spatial correlation in our solution.

In addition, we can build a table which determines how much one variable is related to another. The correlation table for variables from real data trace is shown in the next section. Coefficient *r* is used to identify what variable is more correlated to another. This highly correlated variable was used to calculate β and α coefficients of the multiple linear regression and also for data recovery in the sink to which the data was not sent.

### 4.2. Simple Linear Regression

The current solutions of data reduction by means of linear regression are performed by using simple linear regression based on the least squares [Equations (2) and (3)], as applied by Matos *et al.* [7]. In that case, each sensor node calculates β and α coefficients by using one variable, usually the epoch/time. Then, the sensor node sends its β and α coefficients to the sink, instead of sending the readings. The advantage of this solution is that energy consumption is reduced, but on the other hand, the prediction is not always accurate.

Two application versions based on simple linear regression (as the current solutions) were developed to compare the performance evaluation of our solution, which use prediction based on univariate correlation (simple linear regression based on the least squares). One application version is also used by Matos *et al.* [7], which uses time as independent variable and based on simple linear regression. Another application version uses temperature as independent variable and is also based on simple linear regression. Coefficients β and α are calculated according to Equations (2) and (3), as follows:

(2) $$\beta =\frac{\sum_{i=1}^{n}\left(x_{i}-\bar{X}\right)*\left(y_{i}-\bar{Y}\right)}{\sum_{i=1}^{n}{\left(x_{i}-\bar{X}\right)}^{2}}$$

(3) $$\alpha =\bar{Y}-\beta *\bar{X}$$

where β represents a constant that is multiplied by the value of each independent variable. α is a constant added to the previous multiplication, resulting in the predicted value. *X* and *Y* are two one-dimensional vectors, which respectively represent samples window of the independent and dependent variables, with *X* = *x*_1_, … ,*x_i_* and *Y* = *y*, …,*y_i_*, where *i* = 1, … ,*n* and *n* is the number of samples. *X̄* and *Ȳ* represent the average of samples of each vector.

Coefficients β and α are calculated by each sensor node and, when arriving at the sink, they are used for data recovery, according to Equation (4):

(4) $$Y_{q_{i}}=\alpha +\beta *X_{p_{i}}$$

where *Y*_*q*_*i*__ and *X*_*p*_*i*__ represent one one-dimensional vectors, which respectively contain the values of the predictions made by one dependent variable *q* and samples window of one independent variable *p*, respectively. *Y*_*q*_*i*__ = *y*_*q*_1__, …,*y*_*q*_*i*__ and *X*_*p*_*i*__ = *x*_*p*_1__, … ,*x*_*p*_*i*__, where *i* = 1, …,*n* and *n* is the number of samples. β and α respectively represent the coefficients calculated by Equations (2) and (3).

This approach is used in current solutions, but we propose the use of multiple linear regression instead of simple linear regression due to the fact that prediction accuracy in multivariate correlation is better. In the next section, we describe how to calculate β and α coefficients to perform our method.

## 5. Proposed Solution

The purpose of our approach is to improve prediction accuracy in the WSN data reduction. We use multivariate correlation to decrease prediction errors by means of multiple linear regression as follows:

1. multivariate temporal correlation is applied to perform prediction of consecutive readings by means of multiple linear regression in each sensor node;
2. each sensor node calculates its β and α coefficients and sends them to the sink, instead of sending all field readings;
3. multivariate spatial correlation is used to detect data overlapping by means of Euclidean distance. Therefore, we avoid that the same information is sent by several neighbor sensor nodes; and
4. the missing data can be generated by the sink.

The main contributions of this paper are: (1) discussion about prediction accuracy in environmental monitoring, which includes the correlation between gathered variables such as temperature, humidity and light; (2) it highlights that it is possible to use more accurate prediction solutions through the multivariate correlation method; and (3) it presents the challenges and shows, in details, the steps required to use this solution for data reduction based on prediction approach by multiple linear regression.

### 5.1. Proposed Mechanism

**Figure 4.**
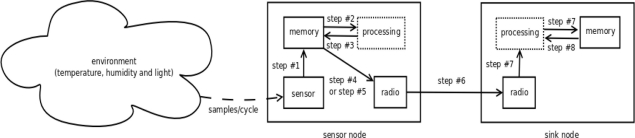
Proposed mechanism diagram.

Our proposed solution is done in eight steps. Some premises are assumed, such as a neighbor coefficients table is created in each sensor node when it starts; a coefficients table is created in the sink; every sensor node remains in promiscuous mode and it stores neighbor coefficients; sampling window must be suitable to maximum size of the packet and defined early by the developer. Figure 4 shows the mechanism according to the steps detailed below:

- Step #1: the sensor node stores a fixed number of samples of gathered readings from all the variables in each cycle.
- Step #2: each sensor node calculates coefficients β and α of the multiple linear regression function when the sampling window reaches the maximum storage threshold previously defined.
- Step #3: before sending its β and α coefficients to the sink, the sensor node looks for duplicated entry in its neighbor coefficients table. These coefficients are received from its neighbor sensor nodes by broadcast.
- Step #4: if the values generated by the sensor node have already been sent to a neighbor sensor node, the sensor node drops its β and α coefficients. Then, it sends a special packet of reduced size, named correlation packet. This packet advertises that the sensor node is correlated to another neighbor sensor node.
- Step #5: if coefficients β and α have not been sent yet by another neighbor sensor node, the sensor node sends them to its parent node until the sink is reached.
- Step #6: the sensor node also sends the sequence of variable readings which is used as independent variable. It is worth mentioning that this variable is calculated by using Pearson’s coefficient [Equation (2)]. In our experiment the independent variable is the temperature.
- Step #7: when coefficients β and α reach the sink, they are used in the multiple linear regression function to predict the readings which have not been sent. Moreover, these coefficients are stored for later use by the correlation packets (Step #4).
- Step #8: if a correlation packet reaches the sink instead of the coefficients, the sink looks for entries from the correlated node in its coefficients table (Step #7). Then β and α coefficients previously stored, are used to predict the readings.

### 5.2. Multivariate Spatial Correlation

WSNs consist of multiple nodes spread in a redundant way. Thus, we get a fault tolerant system through dense networks. On the other hand, these networks are usually composed of resource constrained devices. The energy is supplied by batteries and energy consumption can be better managed when the correlations from monitoring applications are taken. Therefore, we can develop solutions which reduce data traffic in the network. The spatial correlation can be exploited to optimize data communication to the sink and between neighbor sensor nodes [2,12,14].

The spatial correlation happens due to similarities of data being sent to the sink by several sources from high density network [2]. As mentioned in the previous section, Pearson’s coefficient [Equation (1)] does not calculate the multivariate spatial correlation. We propose the use of the Euclidean distance to determine the multivariate spatial correlation between two multidimensional vectors, instead of using Pearson’s coefficient. The Euclidian distance shows how close a multidimensional vector is to another. The Euclidian distance is defined as follows:

(5) $$d_{X_{N},X_{V}}=\sqrt{\sum_{j=1}^{k}{\left(x_{N_{j}}-x_{V_{j}}\right)}^{2}}$$

where *X*_*N*_ = *x*_*N*_1__, … ,*x*_*N*_*j*__ and *X*_*V*_ = *x*_*V*_1__, … ,*x*_*V*_*j*__. In our case *d*_*X*_*N*_, *X*_*V*__ represents the correlation between two multidimensional vectors of dimension *k* with *j* = 1, … ,*k* to be compared in terms of their correlations. Each vector contains the values of β and α coefficients of each gathered variable by sensor node *N* and its neighbor sensor node *V*.

The smaller the Euclidean distance is, the greater is the correlation between two vectors. Thus, we can compare coefficients β and α of the multiple linear regression generated from consecutive readings gathered by a sensor node to β and α coefficients from its neighbor sensor nodes at a given time. The sensor node checks if there is correlation between itself and its neighbor sensor nodes (Step #3), before sending a packet containing β and α coefficients of the multiple linear regression function. If the Euclidian distance is close to 0 (zero), then it means that a packet with the same content was previously sent by any other neighbor sensor node (Step #4).

In our proposed solution, the sensor node detects if there is multivariate spatial correlation between itself and its neighbor node by tree-based routing. This is similar to the compression mechanism adopted by Li *et al.* [8]. The sensor node checks the relationship degree of coefficients β and α by calculating the value of *d*_*X*_*N*_, *X*_*V*__ [Equation (5)].

The sensor node does not send coefficients β and α of the current readings to the sink if the Euclidian distance is 0 (zero). It eliminates the overlapping of information between neighbor sensor nodes. Thus, some sensor nodes do not send data packets at a given time. Therefore, it reduces the broadcast between neighbor sensor nodes and also the data forwarded by the relays.

### 5.3. Multivariate Temporal Correlation

The temporal correlation happens due to the fact that the sensor node gathers correlated data from one or more variables at a given time. This type of correlation is observed due to the nature of physical phenomena [2] (e.g., the environment temperature changes slowly according to time). The simple linear regression function is able to work over temporal correlation, but it is not able to work over the multivariate temporal correlation (more than one variable). We propose the use of multiple linear regression function to work over the multivariate correlation.

Our data reduction solution occurs in a distributed way, where each sensor node calculates coefficients β and α from the multiple linear regression function (Step #2). Then, it only sends β and α if there is no multivariate spatial correlation with other neighbor sensor node.

Coefficients β and α are not calculated by the simple linear regression as the amount of independent variables is greater. The multiple linear regression is described below:

$$\beta =\left(\begin{array}{c} \beta_{0}\\ \beta_{1}\\ \vdots \\ \beta_{j} \end{array}\right),X^{\prime }=\left(\begin{array}{cccc} 1 & 1 & \cdots & 1\\ x_{11} & x_{12} & \cdots & x_{1i}\\ \vdots & \vdots & \vdots & \vdots \\ x_{j1} & x_{j2} & \ldots & x_{\mathrm{ji}} \end{array}\right),X=\left(\begin{array}{cccc} 1 & x_{11} & \cdots & x_{1j}\\ 1 & x_{21} & \cdots & x_{2j}\\ \vdots & \vdots & \vdots & \vdots \\ 1 & x_{i1} & \cdots & x_{\mathrm{ij}} \end{array}\right)\;\text{and}Y=\left(\begin{array}{c} y_{0}\\ y_{1}\\ \vdots \\ y_{i} \end{array}\right)$$

with

(6) $$\beta ={\left(X^{\prime }X\right)}^{-1}X^{\prime }Y$$

where β represents the vector of coefficients of the multiple linear regression function. We use β_0_ = α for simplicity and compatibility with β and α coefficients of the simple linear regression. *X* is one multidimensional vector, which represents the samples window of the independent variable, together with its transpose vector *X*′. *Y* is the one-dimensional vector, which represents the samples window of the dependent variable. *i* = 1, … ,*n* and *n* is the number of samples, and *j* = 1, …, *k* where *k* is the dimension of vector *X*.

### 5.4. Data Recovery

The sink receives β and α coefficients, or the correlated packet for data recovery by means of prediction. It distinguishes this based on packet size. Thereafter, the predictor calculates the values of the missing readings based on β and α coefficients of the multiple linear regression function [Equation (6)]. However, if the correlated packet arrives at the sink instead of coefficients, it uses β and α coefficients of the correlated sensor node stored in the coefficients table of the sink.

In our approach, we decided to adopt a statistical technique to be the predictor due to two main reasons: (1) we are initially developing studies to assess the effects of multivariate correlation and its advantages over univariate correlation; and (2) we intend to adopt computational intelligence techniques to identify its benefits over statistical techniques in further works.

The prediction of variables using multiple linear regression is calculated according to Equation (7):

(7) $$Y_{q_{\mathrm{ij}}}=\beta_{0}+\beta_{1}*X_{p_{i1}}+,\ldots ,+\beta_{j}*X_{p_{\mathrm{ij}}}$$

where *Y*_*q*_*ij*__ represents one one-dimensional vector, which contains the values of predictions made for one dependent variable *q* and *X*_*p*_*ij*__ represents the multidimensional vector, which contains values history of the samples from more than one independent variable *p*. *Y*_*q*_*ij*__ = *y*_*q*_*i*1__, … ,*y*_*q*_*ij*__ and *X*_*p*_*ij*__ = *x*_*p*_*i*1__, …, *x*_*p*_*ij*__, with *i* = 1, … ,*n*, where *n* is the number of samples, and *j* = 1, …, *k*, where *k* is the dimension of vector *X*_*p*_*ij*__. β and α respectively represent the coefficients calculated using Equation (6). As a reminder, β_0_ = α due to compatibility with the notation of β and α coefficients used in this paper.

The prediction by simple linear regression is calculated by Equation (4), but our proposed solution uses a multivariate correlation, instead of a univariate one. Then, our solution uses Equation (7) to perform predictions of the values of the variables.

## 6. Methodology

We used simulation to prove the performance of our solution. The simulation tool adopted was Tossim (http://docs.tinyos.net/tinywiki/index.php/TOSSIM), because we have the device kits of Crossbow (http://www.xbow.com) to later perform testbeds on field and improve our solution. This kind of device supports TinyOS 2.x and Tossim is the default tool to do the simulations.

The whole code was developed for simulation by nesC to TinyOS 2.x. They can be embedded within the sensor nodes of the Tossim simulator and also within the real sensor nodes. This ensures that the same code used to simulate the experiments is able to perform tests in real scenarios in the future.

The simulation scenarios involve different situations of network density, data application values (gathered variables, correlated or not) and way of node deployment. Thus, we check possible real word scenarios by simulation.

Application versions were created to check the improvement of our solution. The first version is the baseline to compare the energy consumption. The aim of this version is to measure the energy consumption without prediction and to check how much each prediction solution will waste when data reduction is used by simple or multiple linear regressions.

The second version is a version adopted by Matos *et al.* [7] to perform data reduction by using simple linear regression. It is a basic prediction version in which we check the prediction errors and energy consumption. This version is based on the time variable, which is not highly correlated with the gathered variables. Therefore, we believe that prediction error tends to increase.

The third version is a way to check if it is possible to improve prediction accuracy by changing only the independent variable. We used the temperature variable instead of the time variable, because it is more correlated with other variables. The best way to improve prediction accuracy is by decreasing prediction errors, using the same energy amount than the second version, but there is a trade-off between prediction accuracy and energy consumption.

The last version is our solution which uses the time and temperature variables together in the prediction. The correlation between gathered variables is higher than the time variable, and then we believe that prediction error will decrease, even though it wastes more energy. Each application version has different packets length, which determines how much energy will be wasted in data communication, *i.e.*, the larger the packet, the greater the energy consumption.

## 7. Performance Evaluation

The performance evaluation was done through four application versions, which we used to simulate and compare multiple linear regression to simple linear regression and to the original version of a monitoring application. This monitoring application simulates the gathering of three variables from the environment: temperature, humidity and light. The application versions to achieve the simulations are:

- First version: original application version, which sends temperature, humidity and light readings periodically every 1,024 clock shots from the sensor node, without performing prediction. This version was created to serve as a reference application for us to compare the energy consumption in the later versions, which uses prediction for data reduction.
- Second version: enhanced version of the original application through a simple linear regression model. It sends only β and α coefficients for each dependent variable. It uses a counter (time variable) as independent variable to predict temperature, humidity and light. This version was designed to verify the energy consumption when simple linear regression is used to reduce data sent to the sink. It was also implemented to calculate SSerr and R^2^ to compare to the next versions. The counter is used as time variable, so it does not send any variable samples to the sink. This version is based on the method proposed by current works as Matos *et al.* [7].
- Third version: enhanced version of the original application through a simple linear regression function, but using the temperature as independent variable, instead of time variable. It sends reading samples of the temperature variable and the β and α coefficients for each dependent variable (except temperature) to predict the dependent variables humidity and light. This version was designed to verify the impact of this model on energy consumption when simple linear regression was sending an independent variable to reduce data communication. It was also created to check SSerr and R^2^ compared to the second and third versions. The temperature was chosen as independent variable due to the results obtained from coefficient *r*, which can be seen later in the next section.
- Fourth version: enhanced version of the original application through a multiple linear regression function, using counter and temperature as independent variables. It sends reading samples of temperature and β and α coefficients for each dependent variable (except temperature) with *β* = (*β*_0_,*β*_1_,*β*_2_) where *α* = *β*_0_. It predicts the dependent variables light and humidity. This version was designed to verify SSerr and R^2^ compared to the second and third versions. Our proposed method is based on this version.

### 7.1. Implementation

**Figure 5.**
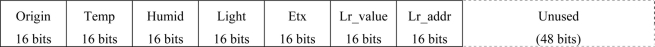
Readings packet length (version 1).

**Figure 6.**
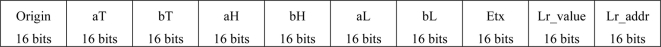
Coefficients packet length (version 2).

**Figure 7.**
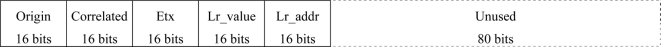
Correlation packet length (version 2).

**Figure 8.**
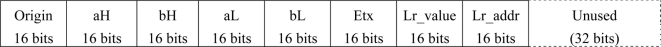
Coefficients packet length (version 3).

**Figure 9.**
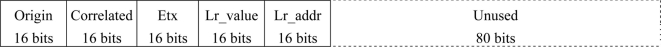
Correlation packet length (version 3).

**Figure 10.**
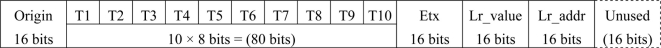
Readings packet length (version 3).

**Figure 11.**
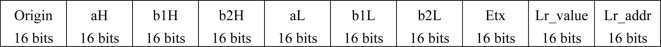
Coefficients packet length (version 4).

**Figure 12.**
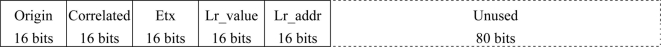
Correlation packet length (version 4).

**Figure 13.**
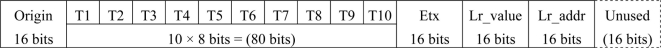
Readings packet length (version 4).

For each application version, we used different types of packet according to each situation. TinyOS 2.x provides, by default, packets up to 28 bytes to be sent by WSN applications, where only 20 bytes can be used by user data and route information. Therefore, we designed application messages with sizes that fit the maximum acceptable size and each version has to be well worked out. The features of each application version are:

- First version: for this version there is only one type of application packet of 14 bytes (Figure 5) containing readings of temperature (Temp), humidity (Humid) and light (Light) variables. The field size of variables is 16 bits due to the fact that data packet in TinyOS does not support float values. Then, to set some variables, such as temperature, the value is converted in integer. In addition to that, this packet contains information to be manipulated by the network layer, such as source node (Origin), route estimation metric (Etx), route value (Lr_value) and next hop (Lr_addr). At each round (cycle) of gathering, a ten readings packet is sent by sensor nodes to the sink, *i.e.*, in the total 140 bytes/round/node.
- Second version: we created two types of application packets: one packet of 20 bytes (Figure 6) containing coefficients β (bT—temperature, bH—humidity and bL—light) and α (aT—temperature, aH—humidity and aL—light) calculated for each dependent variable; and one reduced size packet of 10 bytes (Figure 7) to send the message that the sensor node is spatially correlated to a neighbor sensor node (Correlated). Moreover, the two packets above contain information to be manipulated by the network layer, such as source node (Origin), route estimation metric (Etx), route value (Lr_value) and next hop (Lr_addr). At each round (cycle) of gathering, one coefficients packet or correlation packet is sent by sensor nodes to the sink, *i.e.*, totaling 20 bytes/round/node or 10 bytes/round/node.
- Third version: three types of application packets were created in this version: one packet of 16 bytes (Figure 8) containing coefficients β (bH—humidity and bL—light) and α (aH—humidity and aL—light) calculated for each dependent variable (except the temperature variable); one reduced size packet of 10 bytes (Figure 9) to send the message that the sensor node is spatially correlated to a neighbor sensor node (Correlated); and one packet of 18 bytes (Figure 10) containing 10 readings of temperature (T1 to T10) in sequence to be used in the prediction of the humidity and light variables. In addition, the three packets above contain information to be handled by the network layer, such as source node (Origin), route estimation metric (Etx), route value (Lr_value) and next hop (Lr_addr). The temperature variable is sent in sequence in a single packet, because it is no longer predicted by the sink and is also used to predict the other two variables. The number of readings sent depends on the maximum packet size of the TinyOS. At each round (cycle) of gathering, one coefficients packet and one readings packet, or only one correlation packet is sent by sensor nodes to the sink, *i.e.*, totaling 34 bytes/round/node or 10 bytes/round/node.
- Fourth version: three types of application packets were created in this version: one packet of 20 bytes (Figure 11) containing coefficients β (b1H—humidity and b1L—light, and b2H—humidity and b2L—light) and α (aH—humidity and aL—light) calculated for each dependent variable (except the temperature variable), with *β* = (*β*_0_,*β*_1_,*β*_2_) where *α* = *β*_0_; one reduced size packet of 10 bytes (Figure 12) to send the message that the sensor node is spatially correlated to a neighbor sensor node; and one packet of 18 bytes (Figure 13) containing 10 temperature readings (T1 to T10) in sequence to be used in the prediction of the humidity and light variables. In addition, the three packets above containing information to be manipulated by the network layer, such as source node (Origin), route estimation metric (Etx), route value (Lr_value) and next hop (Lr_addr). The temperature variable is sent in sequence in a same packet as in the third version, because it is no longer predicted by the sink and is also used to predict the other two variables. The number of readings sent depends on the maximum packet size of the TinyOS. At each round (cycle) of gathering, one coefficients packet and one readings packet, or only one correlation packet is sent by sensor nodes to the sink, *i.e.*, totaling 38 bytes/round/node or 10 bytes/round/node.

### 7.2. Simulations Settings

Implemented applications have been run in Tossim. We have used traces (Intel Berkeley Research Lab on http://db.csail.mit.edu/labdata/labdata.html) containing temperature, humidity and light readings gathered by multisensors in a building. Thus, the data gathered for our simulation comes from a scenario close to reality. It contains readings of 54 sensor nodes deployed in laboratories at intervals of 31 seconds. These readings were held during the day, between 28 February and 5 April 2004.

We embed all four application versions within the sensor nodes in the Tossim. Then, the performance of prediction accuracy of the different applications was measured. Also, the energy consumption of data communication in an original application version was tracked. The energy consumption of the original version with three enhanced versions was compared, with two using simple linear regression and one using multiple linear regression (our proposed solution).

The two parameters used to reveal the overperformance or underperformance of prediction accuracy of our solution compared to current works are the Residual Sum of Squares (SSerr) and coefficient of determination (R^2^). SSerr [Equation (8)] is the sum of power of prediction errors for each dependent variable using simple or multiple linear regression. R^2^ [Equation (9)] represents the improvement of the sum of the power of prediction errors. More details about these parameters can be found in Hair *et al.* [9]:

(8) $$\mathrm{SSerr}=\sum_{i=1}^{n}{\left(Y_{i}-Y_{q_{i}}\right)}^{2}$$

Let

$$\mathrm{SSreg}=\sum_{i=1}^{n}{\left(Y_{q_{i}}-\bar{Y}\right)}^{2};\;\;\mathrm{SStot}=\sum_{i=1}^{n}{\left(Y_{i}-\bar{Y}\right)}^{2}$$

(9) $$R^{2}=\frac{\mathrm{SSreg}}{\mathrm{SStot}}$$

where *Y*_*q*_*i*__ represents an one-dimensional vector, which contain the values of the predictions made by one dependent variable *q*. *Y*_*q*_*i*__ = *y*_*q*_1__, …,*y*_*q*_*i*__, where *i* = 1, … ,*n* and *n* is the number of samples. *Y* is an one-dimensional vector, which represents samples window of the independent variables, with *Y* = *y*, … ,*y_i_*, where *i* = 1, …, *n* and *n* is the number of samples. *Ȳ* represents the average of samples of the vector. SSreg is the regression sum of squares and SStot is the total sum of squares.

The performance evaluation of our solution was also measured by ranging the sample amount. This shows how much our solution is affected by the trade-off between prediction accuracy and energy consumption. We repeated the scenario that had the best results among the scenarios simulated to check the behavior of our solution.

### 7.3. Evaluation Metrics

The evaluation metrics adopted for this work are: (1) efficiency of the energy consumption metrics; (2) and efficiency of the predictor metrics. Efficiency of energy consumption metrics are defined as—the total average of energy consumption in the network in Joule from the transmission of application packets (E_trans_); the total average of energy consumption in the network in Joule from the reception of application packets by broadcast of the neighbor sensor nodes—gossiped (E_recp_); the number of times that the multivariate spatial correlation was detected by sensor nodes (C_spatial_); and the percentage of saved energy in the versions with linear regression (versions 2 to 4) in face of the original version (E_saved_). Predictor efficiency metrics are defined as—the prediction error rate (SSerr); and the predictor improvement based on the coefficient of determination (R^2^).

Energy waste in data communication is addressed by the energy consumption metric. According to each application version, the packet length is smaller in initial versions and is bigger in final versions. Thus, the energy consumption tends to be higher in the final version.

The spatial correlation is measured by the amount of times it is detected, showing how an application version saved energy by not sending a large data packet. Perhaps there are no significant differences between the applications versions, since this mechanism has not been modified, but only adapted for each other.

SSerr shows how many errors each application version has over the other. Probably, the initial versions has a higher prediction error than the last versions, because the use of correlated variables in prediction ensures fewer errors.

Coefficient of determination measures the improvement of predictor in relation to its error. Unlike SSerr, the improvement tends to be better in final versions.

Our work aims to improve prediction accuracy and is not more focused on saving energy than current solutions, but nevertheless we have checked the impact of our solution in face of current solutions to measure how feasible it is in a WSN.

### 7.4. Simulation Scenarios

Three characteristics are important to set up scenarios in our simulation. The first one is the behavior of the light variable. Sometimes, the light variable changes easily and leads to different results in the prediction, due to the variation of correlation between gathered variables. It can be presented in two forms, constant and not constant. Temperature and humidity variables are usually correlated, *i.e.*, when one increases the other decreases and vice versa. Therefore, their behavior is constant, with their values changing simultaneously and slowly. Application versions from 2 to 4 use prediction and can increase prediction error when one or more variables change their values quickly.

The second one is the topology which can increase the energy consumption in random deployments. Usually, all application versions suffer the same effects on energy consumption, since the topology will not affect the prediction.

The last one is the network density which also influences the energy consumption, but does not affect the prediction. When the network density is high, *i.e.*, many nodes close to each other, the energy consumption increases due to packet reception by broadcast. The application versions from 2 to 4 should suffer the same effects of network density, but it has to be checked whether the communication between sensor nodes with the lowest prediction error can optimize energy consumption.

Then, in order to explain the simulation scenarios, we summarize the characteristics in Table 2. These characteristics try to emulate the circumstances of the real world so that we can simulate scenarios close to a deployment of sensor nodes for environment applications. We have defined six different scenarios that have been run 30 times each. All scenarios use four application versions and number of nodes ranging from 4 to 100 (to measure scalability). Scalability is important to check energy consumption in all application versions. All scenarios that obtained results from experiments have confidence interval of 95%.

The Link Layer Model tool of TinyOS 2.x was used to create the grid and random topologies. In each scenario several nodes densities are used and summarized in Table 3. The energy consumption model adopted is the same of Jurdak *et al.* [18], where the radio spends 1.67 μJ/Byte sending and 1.89 μJ/Byte receiving data by using micaz mote from Crossbow.

## 8. Simulation Results

### 8.1. Evaluation of the Correlation Analysis

The coefficient *r* results (Table 4) show that there is a greater correlation between the temperature variable and other variables gathered by the sensor nodes (such as humidity and light) than with the time variable. The time variable is the usual variable used in state of art examples.

Given this, the temperature variable was used as independent variable for the application versions 3 and 4. Application version 2 uses only the time variable as independent variable and application version 3 uses only the temperature variable as independent variable, instead of the time variable. On the other hand, application version 4 uses the time variable and temperature variable as independent variables.

### 8.2. Energy Consumption

The main goal of our proposed solution is not to reduce energy consumption compared to the existent approaches based on simple linear regression, but rather find the best trade-off between energy consumption and prediction accuracy. In our method we use samples of the temperature variable to predict the humidity and light variables. While we slightly increase energy consumption compared to simple linear regression, we improve the prediction accuracy caused by simple linear regression.

**Figure 14.**
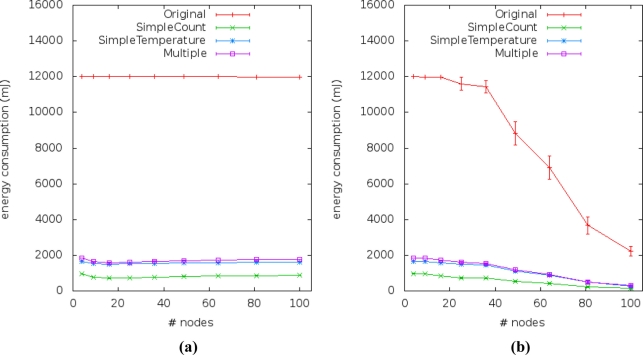
Average energy of the radio consumed by messages sent to the sink: **(a)** Scenarios #1, #2, #5 and #6. **(b)** Scenarios #3 and #4.

**Figure 15.**
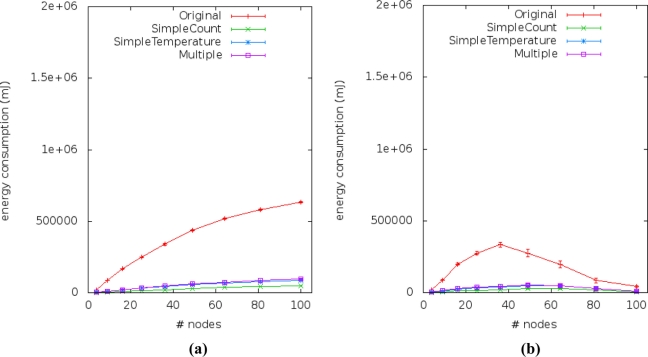

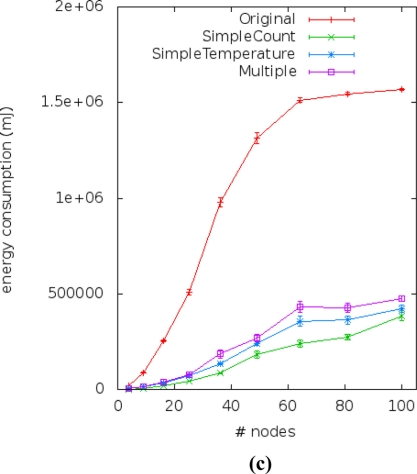
Average energy of the radio consumed by messages received for gossip routing: **(a)** Scenarios #1 and #2. **(b)** Scenarios #3 and #4. **(c)** Scenarios #5 and #6.

Figures 14 and 15 show the energy consumption results obtained from simulations of the four application versions. They describe the performance of energy consumption for transmission (E_trans_) and reception (E_recp_) of data by sensor nodes. We observed the impact of our method by comparing the energy consumption of the multiple linear regression (our solution) to the simple linear regression (current works).

Under all conditions, the energy consumption is greater in the application versions that use simple or multiple linear regression based on the temperature variable instead of the time variable. This happens because when using the independent variable gathered by the sensor nodes, their reading samples have to be sent to the sink. Hence, they consume more energy than the application version that uses time (the counter) as independent variable.

The energy consumption due to message exchanges between sensor nodes in scenarios #1, #2, #5 and #6 is presented in Figure 14(a). The E_trans_ relation between the approaches remains constant, even when scalability changes and the approaches which use gathered variables consume twice as much E_trans_ than approaches which not use it. The relation between the E_trans_ of the original application and approaches with gathered variable is about 0.17 and with the current approach is about 0.08. In scenarios #3 and #4, the communication failure affected the energy consumption [Figure 14(b)] of all application versions when density falls below 0.0278 (from 36 to 100 sensor nodes).

We checked that the energy consumption of the data sent by sensor nodes in the second application version (a.k.a. SimpleCount) is the lowest [Figure 14(a,b)], due to the fact that this application does not send reading samples to the sink. This application is the one adopted by current approaches.

Nevertheless, we can also see that the energy consumptions of the third and fourth application versions (a.k.a. SimpleTemperature and Multiple, respectively) are the closest to the SimpleCount in face of the first application version (a.k.a. Original). Thus, it appears as stated before that our solution uses double the energy of the current solutions, but its energy consumption is still low when compared to the version without prediction (original version).

The amount of energy spent to receive messages (E_recp_) from application broadcast on the transmission of neighbor sensor nodes (routing gossip) is observed in Figure 15. In some scenarios [Figure 15(c)], the E_recp_ of our approach is about three times smaller than the original application, but still consuming more energy than the current approach. We can see more details of the percentage of energy saving from the three application versions that use simple or multiple linear regression in face of the original application version in Table 5.

The results of spatial correlation (C_spacial_) showed no differences between our approach and current approaches, but it points to the fact that is essential to save energy. The amount of times that the correlation was detected is greater in the scenarios where there is fixed density of 0.25 sensor nodes per m^2^, *i.e.*, in scenarios #5 and #6. It shows that in higher density situations the packets will not be sent twice to the sink. Thus, we avoid overlapping and save more energy.

### 8.3. Performance Evaluation of the Prediction Accuracy

**Figure 16.**
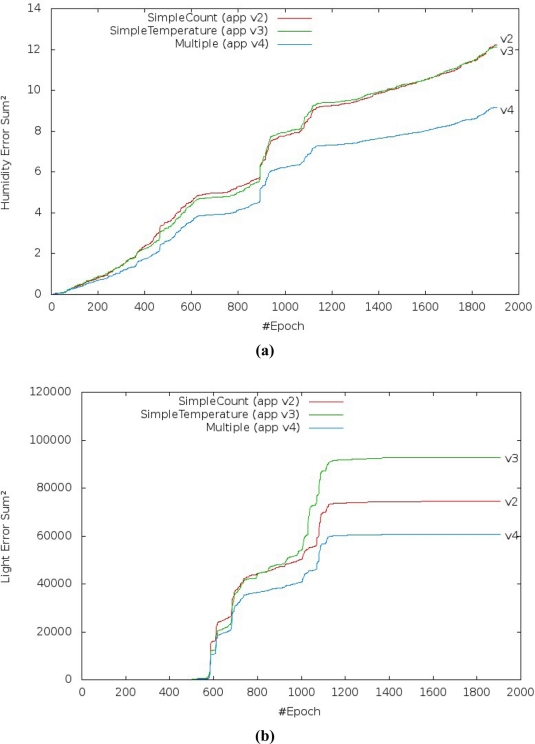

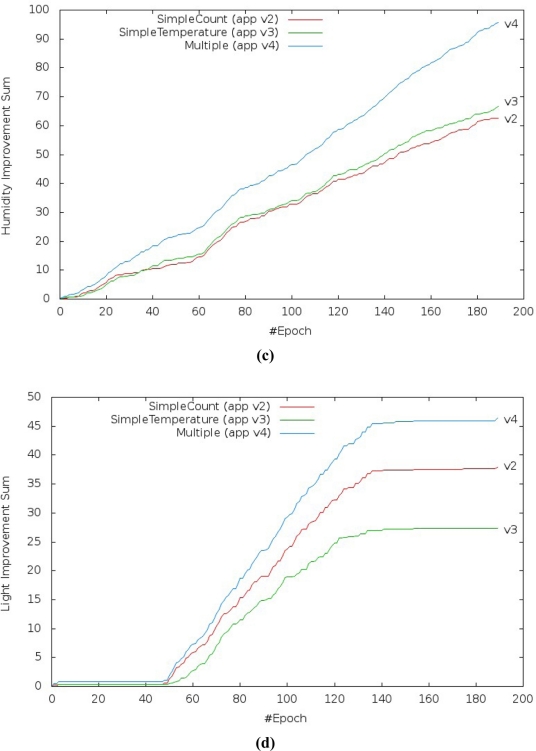
Performance evaluation of the prediction accuracy over one day from the trace to the application versions which use linear regression (app v2 to app v4): **(a)** Humidity error. **(b)** Light error. **(c)** Humidity improvement. **(d)** Light improvement.

Figure 16 shows the prediction performance of the three application versions which use linear regression over one day of data gathering from the Intel Research Lab’s trace. The error and improvement performance to the humidity and light variables ensures that our solution is better than current solutions.

The SSerr and R^2^ results from prediction of humidity [Figure 16(a,c)] show, for all scenarios, that the lowest prediction accuracy was obtained when we compared simple linear regression based on the time and temperature variables as explanatory variable. The best prediction accuracy was obtained when multiple linear regression was used. However, energy consumption is higher in the versions that use simple or multiple linear regression based on the temperature variable instead of the time variable, although they still get better values than the original version.

The SSerr and R^2^ results from prediction of the light [Figure 16(b,d)] show for all scenarios that the highest prediction error was obtained when we compared simple linear regression based on the time and temperature variables as explanatory variable. The lowest prediction error was obtained when multiple linear regression was used.

We also observed that there are different behaviors in the results [Figure 16(b,d)] where the light variable is irregular. As per the previous section, the gathered readings of the light variable in the trace are irregular, *i.e.*, the values in the trace do not follow a sequence (increasing or decreasing). This probably denotes noise or on and off procedures, and high sensitivity of the light sensor. Thus, the results of the prediction of the light variable show the drawback of multiple linear regression, although it still gets better results than the current approach. When there is no correlation between the variables, prediction accuracy decreases or does not work properly.

Therefore, we suggest that by using prediction based on multiple linear regression, the sensor node checks the improvements in an adaptive way, as in Jiang *et al.* [11]. Tables 6 and 7 show more details of the results of SSerr and R^2^.

### 8.4. Trade-Offs of Our Solution

After the results above, we decided to repeat the simulation to evaluate the energy consumption and prediction accuracy performance and analyze the behavior of our solution. The trade-off between these two performances is intrinsic because, in order to increase prediction accuracy, our solution sends samples gathered from a variable. Therefore, our solution consumes more energy than current solutions.

The relationship between energy consumption and prediction accuracy does not depend on the amount of sensor nodes, because prediction is done in a distributed and localized way. We learned that it depends on the amount of samples. Therefore, when we increase the amount of samples, energy consumption decreases, SSerr increases and R^2^ decreases, but the WSN cannot spend much energy, thus scenario #6 was simulated again, due to the fact that it had better performance results than the other scenarios.

The amount of samples ranged from 6 (six), 8 (eight) and 10 (ten), which we respectively named Scenario #6C, Scenario #6B and Scenario #6A. The energy consumption results in these scenarios from messages sent by the sensor nodes show that, in order to decrease the amount of samples from 10 (Scenario #6A with 100 sensor nodes) to 6 (Scenario #6C with 100 sensor nodes), the E_trans_ of the network increased from 1,834.32 μJ to 2,465.70 μJ. This happens because, by reducing the amount of samples, more packets will be sent. The E_recp_ results show that the energy consumption increased from 489,567.40 μJ (Scenario #6A with 100 sensor nodes) to 578,866.80 μJ (Scenario #6C with 100 sensor nodes).

**Figure 17.**
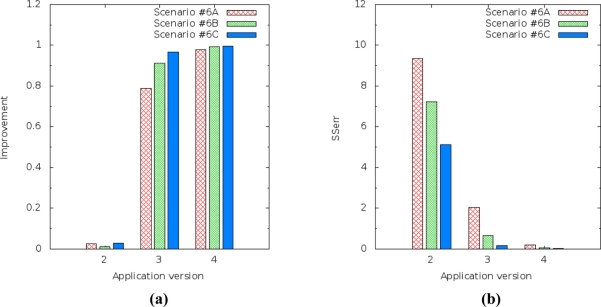
Improvement and SSerr of the prediction performed by application versions for the humidity variable ranging sample amount (Scenario #6A—ten samples, Scenario #6B—eight samples and Scenario #6C—six samples): **(a)** Improvement for humidity. **(b)** SSerr for humidity.

The prediction improvement of humidity for the application version 4 (multiple linear regression) decreased from 0.995868 to 0.978811 [Figure 17(a)] and the SSerr of humidity increased from 0.021840 to 0.203488 [Figure 17(b)]. It should also be noted that application version 4 always had better results than the others versions.

**Figure 18.**
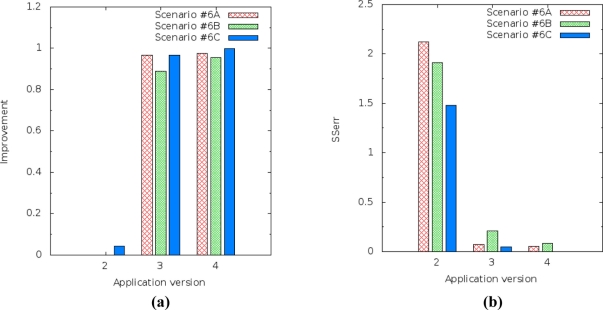
Improvement and SSerr of the prediction performed by application versions for the light variable ranging sample amount (Scenario #6A—ten samples, Scenario #6B—eight samples and Scenario #6C—six samples): **(a)** Improvement for light. **(b)** SSerr for light.

The results for light level prediction are a little bit different from the results for humidity, but they display the same behavior. The improvement of the light level prediction for application version 4 (multiple linear regression) decreased from 0.999752 to 0.974384 [Figure 18(a)] and the SSerr of the light increased from 0.000384 to 0.054342 [Figure 18(b)].

**Figure 19.**
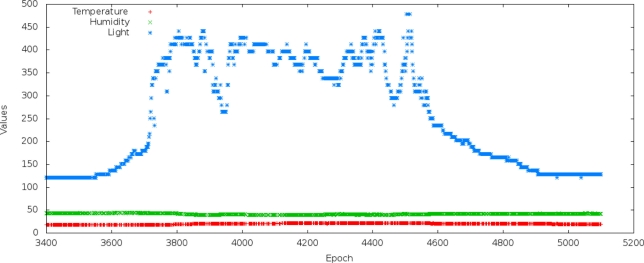
Epochs from a collect day where the light variable is less correlated with the temperature and humidity variables.

The results obtained from light variable prediction were different from the results obtained from humidity variable prediction. Then, we checked the behavior of the threes gathered variables and used them in our performance evaluation. Figure 19 shows epochs from a data collection day where the correlation between the variables is low. Note that in epochs ranging from 3,550 to 4,900, the light variable increases a lot. Consequently, the simple and multiple linear regressions tend to worsen prediction accuracy. This explains some abnormal results when we used the light variable as independent variable.

## 9. Discussion and Conclusions

Several sensor boards are able to monitor more than one variable (multisensor), adding new challenges, such as increasing precision by reducing prediction error. In this paper, we propose a method to improve prediction accuracy in WSN data reduction by applying multivariate spatial and temporal correlations.

Prediction accuracy of correlation mechanisms depends on the correlation analysis to determine which variable is highly correlated. The current approaches are not focused on the analysis of correlations and hence the prediction errors tend to be higher. The correlation analysis results of Table 2 show that the time variable is the least correlated of all others. Thus, predictions using weakly correlated variables can greatly increase errors. We recommend that all proposals contemplate the analysis of correlation to obtain better results in their predictions. Although energy consumption of our solution is twice that of the simple linear regression approach, it is still smaller than the original application. Table 5 summarizes the energy savings for the application versions that perform prediction and shows that the version using simple linear regression is the most economical and multiple linear regression is the one that consumes more energy. An important observation is that the simple linear regression, using the variable temperature as independent variable, is not the best option to improve prediction, although it spends a little more energy than the version with multiple linear regression, the first does not show better accuracy results than the second.

Related works use simple linear regression based on the time variable as independent variable, so that they are more susceptible to errors than our proposal. Although multiple linear regression spends more energy than simple linear regression, it may be the best choice, especially for accuracy-sensitive applications (e.g., precision agriculture).

We conducted simulations involving simple and multiple linear regression functions (application versions from 2 to 4) to assess our prediction solution. The values of residual sum of squares (SSerr) and coefficient of determination (R^2^) show that prediction accuracy may be the lowest, where simple linear regression based on the time variable is used as explanatory variable. Also, these results show that the best prediction accuracy is obtained when multiple linear regression is used. The multivariate correlation method outperforms some current methods in about 50% to humidity prediction and 21% to light prediction.

Table 6 shows that predictions were more accurate when our solution was used, because when more than one variable is used in the prediction, error decreased, but Table 7 shows the disadvantage of our solution, because when the variables are not strongly correlated, the prediction error tends to be higher than in solutions that use simple linear regression, e.g., when the light variable is not constant.

Finally, we have done some works trying to improve WSN solutions [19–22] and intend to further reduce energy consumption considering sensing, processing and communication. Computer intelligence algorithms [23] and cluster routing solutions [19] may be helpful in better adapting our spatio-temporal correlation solution to improve network lifetime.

## Figures and Tables

**Table 1.** Comparison of the main characteristics of solutions.

**Work**	**Main Characteristics**					
	**Topology**	**Spatial Correl.**	**Temporal Correl.**	**Mechanism**	**Multivariate**	**Correlation Analysis**
Goel and Imielinski [10]	Centralized	Yes	No	MPEG Standard—like	No	No
Xu and Lee [15]	Localized	Yes	Yes	Dual prediction	No	No
Matos *et al.* [7]	Distributed	No	Yes	Simple Linear Regression	No	No
Silva *et al.* [17]	Distributed	No	Yes	Principal Component Analysis	No	No
Our solution	Distributed	Yes	Yes	Multiple Linear Regression	Yes	Yes

**Table 2.** Characteristics of the simulation scenarios.

	**Features**						

	**Light variable**		**Topology**		**Network density**		

**Scenarios**	**Constant**	**Not constant**	**Grid**	**Random**	**1 node/5 m**	**Ranging**	**Fixed**
1	X		X		X		
2		X	X		X		
3		X		X		X	
4	X			X		X	
5		X		X			X
6	X			X			X

**Table 3.** Network density in the simulation scenarios.

	**Density (nodes/m^2^) by scenarios**					
**Nodes**	**#1**	**#2**	**#3**	**#4**	**#5**	**#6**
4	0.1600	0.1600	0.2500	0.2500	0.2500	0.2500
9	0.0900	0.0900	0.1111	0.1111	0.2500	0.2500
16	0.0711	0.0711	0.0625	0.0625	0.2500	0.2500
25	0.0625	0.0625	0.0400	0.0400	0.2500	0.2500
36	0.0576	0.0576	0.0278	0.0278	0.2500	0.2500
49	0.0544	0.0544	0.0204	0.0204	0.2500	0.2500
64	0.0522	0.0522	0.0156	0.0156	0.2500	0.2500
81	0.0506	0.0506	0.0123	0.0123	0.2500	0.2500
100	0.0494	0.0494	0.0100	0.0100	0.2500	0.2500

**Table 4.** Results of the correlation analysis.

	**Temperature**	**Humidity**	**Light**	**Time**
**Temperature**	1.0000	−0.7987	0.4550	−0.2681
**Humidity**	−0.7987	1.0000	−0.2489	0.1987
**Light**	0.4550	−0.2489	1.0000	−0.1807
**Time**	−0.2681	0.1987	−0.1807	1.0000

**Table 5.** Percentage of the energy saving for sending and receiving data in face of the original application version.

**App. version**	**Scenario #1**		**Scenario #2**		**Scenario #3**		**Scenario #4**		**Scenario #5**		**Scenario #6**	

	**Sent**	**Gossiped**	**Sent**	**Gossiped**	**Sent**	**Gossiped**	**Sent**	**Gossiped**	**Sent**	**Gossiped**	**Sent**	**Gossiped**
2	0.93	0.93	0.93	0.93	0.93	0.89	0.93	0.92	0.92	0.87	0.92	0.87
3	0.87	0.87	0.87	0.87	0.87	0.82	0.87	0.85	0.86	0.82	0.86	0.82
4	0.86	0.86	0.86	0.86	0.86	0.81	0.86	0.84	0.85	0.79	0.85	0.80

**Table 6.** Performance results of the SSerr and R^2^ from all versions in scenarios #1, #4 and #6.

	**Independent variable**					

	**Count (Time)**		**Temperature**		**Count and Temperature**	

	**version 2**		**version 3**		**version 4**	

	**SSerr**	**R^2^**	**Sserr**	**R^2^**	**SSerr**	**R^2^**
Temperature	0.210300	0.296891	−	−	−	−
Humidity	9.355700	0.025813	2.033940	0.788210	0.203488	0.978811
Light	2.121380	0.000000	0.073135	0.965525	0.054342	0.974384

**Table 7.** Performance results of the SSerr and R^2^ from all versions in scenarios #2, #3 and #5.

	**Independent variable**					

	**Count (Time)**		**Temperature**		**Count and Temperature**	

	**version 2**		**version 3**		**version 4**	

	**SSerr**	**R^2^**	**Sserr**	**R^2^**	**SSerr**	**R^2^**
Temperature	10.321800	0.290535	−	−	−	−
Humidity	4.964100	0.476813	8.583820	0.095316	0.185308	0.980470
Light	140.150060	0.869629	794.135000	0.261311	1075.060000	0.000000
